# The Adaptive GameSquad Xbox-Based Physical Activity and Health Coaching Intervention for Youth With Neurodevelopmental and Psychiatric Diagnoses: Pilot Feasibility Study

**DOI:** 10.2196/24566

**Published:** 2021-05-14

**Authors:** April B Bowling, James Slavet, Chelsea Hendrick, Robbie Beyl, Phillip Nauta, Marilyn Augustyn, Mediatrix Mbamalu, Carol Curtin, Linda Bandini, Aviva Must, Amanda E Staiano

**Affiliations:** 1 Department of Public Health and Nutrition School of Health Sciences Merrimack College North Andover, MA United States; 2 Marblehead Public Schools Marblehead, MA United States; 3 Pennington Biomedical Research Center Baton Rouge, LA United States; 4 Developmental and Behavioral Pediatrics Clinic Boston Medical Center Boston, MA United States; 5 Department of Family Medicine and Community Health Eunice Kennedy Shriver Center University of Massachusetts Medical School Worcester, MA United States; 6 Healthy Weight Research Network Worcester, MA United States; 7 Department of Health Sciences Sargent College of Health and Rehabilitation Sciences Boston University Boston, MA United States; 8 Department of Public Health and Community Medicine Tufts University Boston, MA United States

**Keywords:** exercise, diet, sleep, mental health, children, adolescent, health promotion, telehealth, exergaming

## Abstract

**Background:**

The prevalence of neurodevelopmental and psychiatric diagnoses (NPDs) in youth is increasing, and unhealthy physical activity (PA), diet, screen time, and sleep habits contribute to the chronic disease disparities and behavioral challenges this population experiences.

**Objective:**

This pilot study aims to adapt a proven exergaming and telehealth PA coaching intervention for typically developing youth with overweight or obesity; expand it to address diet, screen, and sleep behaviors; and then test its feasibility and acceptability, including PA engagement, among youth with NPDs.

**Methods:**

Participants (N=23; mean age 15.1 years, SD 1.5; 17 males, 9 people of color) recruited in person from clinic and special education settings were randomized to the Adaptive GameSquad (AGS) intervention or wait-list control. The 10-week adapted intervention included 3 exergaming sessions per week and 6 real-time telehealth coaching sessions. The primary outcomes included feasibility (adherence to planned sessions), engagement (uptake and acceptability as reported on process questionnaires), and PA level (combined light, moderate, and vigorous as measured by accelerometer). Descriptive statistics summarized feasibility and engagement data, whereas paired, two-tailed t tests assessed group differences in pre-post PA.

**Results:**

Of the 6 coaching sessions, AGS participants (n=11; mean age 15.3 years, SD 1.2; 7 males, 4 people of color) completed an average of 5 (83%), averaging 81.2 minutes per week of exergaming. Of 9 participants who completed the exit questionnaire, 6 (67%) reported intention to continue, and 8 (89%) reported feeling that the coaching sessions were helpful. PA and sleep appeared to increase during the course of the intervention over baseline, video game use appeared to decrease, and pre-post intervention PA per day significantly decreased for the control (−58.8 min; *P*=.04) but not for the intervention group (−5.3 min; *P*=.77), despite potential seasonality effects. However, beta testers and some intervention participants indicated a need for reduced complexity of technology and more choice in exergames.

**Conclusions:**

AGS shows promise in delivering a health behavior intervention remotely to youth with NPDs, but a full-scale efficacy trial with a larger sample size is needed to confirm this finding. On the basis of feedback from beta testers and intervention participants, the next steps should include reduced technology burden and increased exergame choice before efficacy testing.

**Trial Registration:**

ClinicalTrials.gov NCT03665415; https://clinicaltrials.gov/ct2/show/NCT03665415.

## Introduction

### Background

Mental health and the resultant adverse chronic disease consequences among youth are growing concerns in the United States. Recent estimates suggest that pediatric psychiatric disorders occur in more than one-fourth of people aged <18 years [1], including diagnoses such as anxiety, mood, and psychotic disorders. Common comorbid diagnoses include neurodevelopmental disorders, such as autism spectrum disorder (ASD) and attention-deficit/hyperactivity disorder (ADHD), which are estimated to affect approximately 15% of US youth [2], leading to significant heterogeneity of symptom presentations.

The health disparities faced by children and youth with heterogeneous neurodevelopmental and psychiatric diagnoses (NPDs) are considerable and include a higher risk of obesity and type 2 diabetes [3], compared with typically developing youth. These disparities are at least partially attributable to unhealthy behavioral patterns established in childhood, patterns that often persist throughout life [4]. Several studies have found that children with ASD; ADHD; and bipolar, depressive, and anxiety disorders are at high risk of low physical activity (PA) levels [5,6], poor diet [7], disrupted sleep [8,9], and elevated screen time [10].

Unhealthy lifestyles among youth with NPDs are doubly unfortunate; they not only confer increased health risks across the life course but also exacerbate the challenges to cognitive and behavioral functioning experienced by this population. More than 25 published studies have documented associations between light-, moderate-, and vigorous-intensity PA and improvements to mood and executive functioning, such as the ability to focus and self-regulate, and meta-cognitive processes among children with NPDs [11]. For example, a recent study found that among children with moderate to severe NPDs who were exposed to cybercycling (ie, stationary bicycles that use immersive gaming features) during the school day, the odds of behavioral dysregulation declined by more than 70% relative to children who did not participate in exercise [12]. These results are consistent with many previous studies showing positive effects of exercise on mood and impulsivity; there is also evidence that these positive benefits may be most pronounced in children with NPDs [13]. Unfortunately, few interventions tested to date have shown effectiveness in increasing long-term engagement in PA among youth with NPDs, nor are many scalable, given their resource intensity.

Exergames have shown promise in promoting cost-effective engagement in light- to moderate-intensity PA in youth [14]. When integrated into theory-based interventions, they may also help improve the personal mediators of exercise engagement in children. For example, youth with ASD and major depressive disorder show high behavioral inhibition bias (BIS) [15], which indicates avoidance of novel or uncomfortable situations and which may act as a barrier to engagement in higher intensities of exercise or new exercise programming [16]. However, previous studies in typically developing youth have shown that acting on constructs of self-determination can help individuals with high BIS adopt long-term intrinsic motivation toward exercise [16]. Tailoring PA interventions to include lower-intensity exercise, agency in selection of exercise intensity, use of fun and noncompetitive exercise technology such as exergames, and monitoring of mood improvements after exercise may be particularly effective.

GameSquad is an intervention originally designed to improve PA among a socioeconomically and racially diverse population of typically developing children who meet the criteria for overweight or obesity [16]. GameSquad is an intervention delivered entirely remotely using exergaming and virtual health coaching (telehealth coaching delivered via video conference) as components within a behavior change intervention grounded in social cognitive theory, which frames behavioral change as the result of links among behaviors, environment, and psychosocial factors [16]. Exergames can be played with family members and friends, and social interaction during group-based exergame play has been identified as a key predictor of weight loss [16]. Exergames also use programmed features that can encourage exercise, such as motivational messaging during game play, to boost players’ self-efficacy, which may translate to self-determination and predict exercise adherence and eventual engagement in nonscreen-based exercise modalities [17]. The GameSquad intervention directly emphasizes the element of social support by encouraging children to play with a family member or friend and by requiring children and parents to attend telehealth counseling sessions together from their home. These coaching sessions are delivered through the gaming console and are designed to promote self-efficacy and teach approaches and strategies to reduce perceived barriers to behavior change. A 6-month randomized controlled trial of the GameSquad intervention found a significant increase in moderate- to vigorous-intensity physical activity (MVPA) and a decrease in BMI, blood lipids, and blood pressure (BP) among participants [16].

### Objective

Inclusion team science is an intervention development and testing framework that brings together disability researchers and intervention scientists to fast-track intervention adaptation for this underserved population [18]. Given GameSquad’s initial efficacy in increasing MVPA in typically developing but difficult to engage youth and the potential of exergaming and virtual health coaching to engage youth with NPDs in positive health behaviors and as a tool to transition them to long-term intrinsic motivation to exercise, the aims of this study are as follows: (1) to adapt this intervention for use among youth with NPDs; (2) to expand the virtual coaching sessions to address dietary habits, screen time, and healthy sleep habits in addition to PA promotion; and (3) to pilot test the initial feasibility, engagement, and short-term changes in health behaviors. The primary outcomes examined during the pilot test included feasibility (adherence), engagement (uptake of PA during the intervention and acceptability), and change in objectively measured PA pre- and postintervention. The secondary outcomes included pre-post changes in self-reported exercise stage of change, sleep duration, hours per week of video game use, problematic mealtime behaviors, BMI, and BP.

## Methods

### Overview

All study procedures were approved by the Merrimack College, Pennington Biomedical Research Center, and Boston Medical Center Institutional Review Boards, and the study was registered as a clinical trial and is available on ClinicalTrials.gov (NCT03665415). As we adapted GameSquad to a population of youth with NPDs to create Adaptive GameSquad (AGS), we expanded the theoretical framework to include the Reserve Capacity Model [19] and the Family Ecological Model [20]. The purpose of this expansion was to ensure that intervention components addressed barriers to and used facilitators of health behaviors specific to the unique challenges faced by this population (eg, depleted caregiver reserve capacity) [21]. Given that remotely delivered home-based interventions are critical for upscaling but can also place a high burden on families, we used these models to act on specific constructs to improve participant and caregiver reserve capacity and downstream health behaviors and outcomes.

Adaptation was undertaken by an advisory team including a developmental psychologist, school psychologist, registered dietitian, clinical social worker, fitness coaches, and parents of a child with NPD. As a result, we expanded caregiver support components, such as scheduling reminders; modified the original challenge booklet to increase MVPA goals more slowly; trained telehealth coaches in positive behavioral reinforcement techniques; and targeted exergames that were particularly engaging for the demographic profile of our target population. We also expanded coaching scripts to not only address PA but also to include health education and goal setting for dietary intake, sleep, and screen time habits. Coaches were also trained to work with youth with NPDs, particularly in positive reinforcement and de-escalation methods [22].

Finally, 3 beta testers and their caregivers were purposively recruited from the school study site for diversity of diagnosis, gender, and grade level (demographic information is not included here to protect them from inferred identification, given the small sample size). After written parental consent and child assent, the youth participated in a 4-week trial of the initial AGS intervention. We then used their feedback to make additional modifications to the telehealth coaching script and gaming menu, including increased agency in game selection and allowing for solitary game play. These modifications resulted in the final AGS intervention deployed for the pilot feasibility and engagement study.

### Recruitment

**Figure 1:**
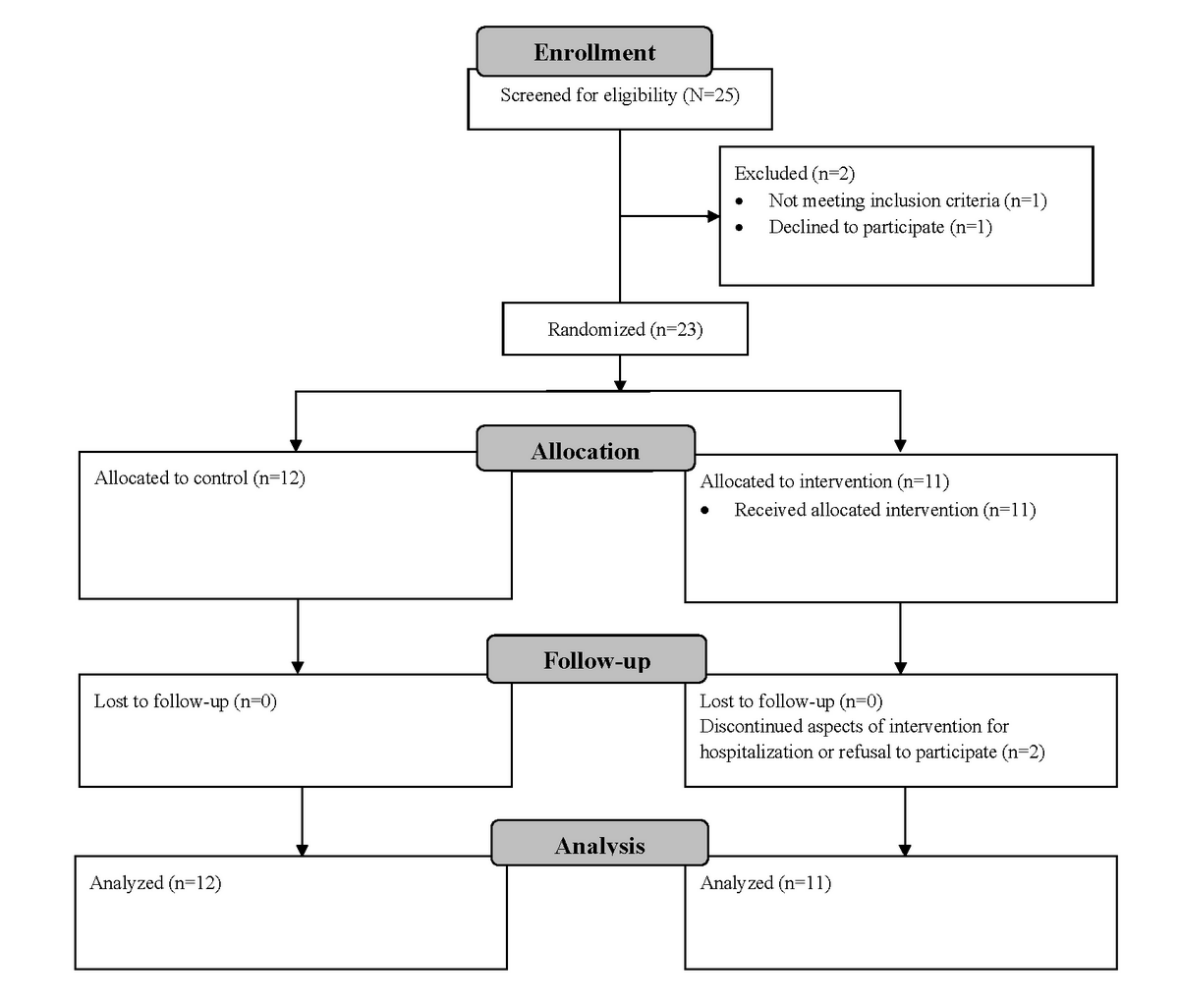
Study recruitment and enrollment flow diagram.

Participants for the 10-week pilot study were recruited in person from October 15, 2018, to February 15, 2019, from either the Boston Medical Center Developmental or Behavioral Pediatrics Clinic from therapeutic programs at a large public middle and high school (Figure 1). This approach helped to ensure a diverse diagnostic sample.

The study was powered as a pilot feasibility and acceptability trial [23,24]. Of the 25 children whose parents completed a web-based screen survey within the recruiting time frame, 23 met the eligibility criteria and were randomly assigned in a 1-to-1 allocation ratio using the REDCap (Research Electronic Data Capture) software. The inclusion criteria included being in middle or high school, having a neurodevelopmental and/or psychiatric diagnosis, willingness to participate in exergaming 3 times weekly and meet with a telehealth coach every other week, and possessing the cognitive ability to understand gaming directions. Exclusion criteria included having impairments that prevented engagement in exergames, pregnancy, inability to speak or understand English, or having a caregiver unable or unwilling to attend the telehealth coaching sessions on a regular basis.

### Procedures

Interested parents who met the criteria during the initial web-based screening then participated in an additional phone screening with research assistants and provided web-based written consent for their child to participate. Eligible children whose parents had consented were then assented in person during the initial data collection visit, during which height, weight, and BP, and psychosocial questionnaire data (see Measures section) were collected. Participants were given and instructed on the use of a hip-worn accelerometer (wGT3X-BT, ActiGraph). These accelerometers do not have digital readouts and thus minimize the effects of measurement on participants’ normal PA patterns. Participants were provided with the accelerometer to wear for 7 days before randomization and again the week after the 10-week intervention, with a follow-up visit scheduled to collect the accelerometer. After the initial wear, the participants were randomized to the condition using the randomization algorithm in REDCap. Participants assigned to the wait-list control condition were asked to maintain their normal level of PA for 10 weeks but did not receive additional information or a PA tracker during the 10 weeks. They then received the same gaming equipment as the intervention participants and 4-week of telehealth coaching after the final data collection. All data assessors and primary investigators were blinded to participant condition. Telehealth coaches and research assistants who installed equipment and training participants were not blinded.

All participants randomized to the intervention group received an Xbox One gaming console with a Kinect motion sensor (Microsoft), a 12-week Xbox Live subscription, and 3 exergames (Just Dance 3, Shape Up 3, and Kinect Sports Season 2). Two research assistants visited intervention participants in their homes to install the equipment and to train participants and caregivers on the use of the games, the Skype portal for coaching sessions, and the Fitbit for tracking of steps during the intervention. The AGS intervention asked participants to play exergames 3 days per week with a family member or friend, if possible (not required). On nonexergaming days, participants were asked to meet tailored and incrementally increasing minutes of PA (starting with 10 min in week 1 and increasing to as much as 40 min per day by week 4). The activities prescribed were laid out week by week in the intervention *challenge booklet* participants were provided. The targeted durations were below PA guidelines [25] but were selected to minimize frustration and improve self-efficacy; participants were also encouraged and worked with coaches to brainstorm nonscreen-based physical activities that they could perform during each week. The challenge booklet provided an adaptable gameplay curriculum to play 3 challenges each week with increasing intensity, difficulty, and duration.

Participants and caregivers were asked to meet with a telehealth coach over a video chat using the Xbox console. Meetings were held in weeks 1, 2, 4, 6, 8, and 10 and were rescheduled as necessary. Participants were asked to wear a Fitbit Charge throughout the 10-week intervention period. They and their caregivers received charging and sync reminders 3 times per week. Steps per day could then be wirelessly and automatically uploaded and reviewed by the telehealth coach. Coaches followed a script for meetings that focused on reviewing the week’s PA, praising progress, troubleshooting barriers, and then discussing a new healthy habit to try each week.

### Measures

Feasibility and engagement measures were tracked by telehealth coaches in REDCap, including telehealth coaching session attendance (coach report), number of exergame sessions completed per week (participant log), minutes of exergaming per week (participant log), and steps per week (Fitbit Charge, Fitabase). We also administered exit surveys with participants and conducted semistructured interviews with beta testers and their caregivers to assess acceptability and elicit suggestions for improvement. Exit surveys were administered after all other follow-up data were collected. Two participants were unavailable for exit surveys; thus, there was a reduced sample size for acceptability measures only.

Participant weight was measured by trained research assistants in light clothing without shoes on a digital scale (Model 813, Seca), and height was measured using a clinical stadiometer (Model 217, Seca). Two measurements were taken for both weight and height, with the average of each used to calculate the BMI (kg/m2). After height and weight were recorded, appropriate cuff size was selected, and diastolic and systolic BP readings were taken, following the 2017 TRUE (International Consortium for Quality Research) Consortium guidelines [26]. To assess intrinsic changes to PA habits outside of active intervention support, time spent in MVPA before the intervention started and 1 week after it concluded was objectively measured with the ActiGraph wgt3x-bt accelerometer. Accelerometers were initialized to record data in 15-second epochs, and established pediatric cutoff points were used to estimate PA of light-, moderate-, and vigorous-intensity levels [27]. We used a 7-day weighted average of weekday and weekend activity counts to determine each participant’s PA level, with a minimum requirement of 600 minutes of wear time that included at least one weekend day [28]. Participants were also asked to keep a log of the number and duration of exergaming sessions they completed per week as part of the challenge booklet they were provided.

Exercise stage of change (youth report) was measured using a pen and paper version of the Change of Stages of Exercise–University of Rhode Island Change Assessment, the third generation [29], a valid and reliable measure that captures both precontemplation nonbelief and belief conditions. Participants also completed a video game use survey, which asked questions regarding their current video game use habits, including hours of nonexergame gaming per week. In addition to a demographic questionnaire, parents were asked to complete the Children’s Sleep Habits Questionnaire [30], which includes duration of sleep, and the Meals In Our Household Questionnaire (MIOH) [31], which measures 6 domains, including the structure of family meals and problematic child mealtime behaviors. We extracted and used the Problem Mealtime Behaviors subscale of the MIOH to evaluate changes related to behaviors targeted by the intervention coaching sessions.

### Statistical Analysis

The sample size was determined on the basis of the aims and design (feasibility and engagement pilot study), with a target of 50 participants but a minimum of 20, based on the current guidelines [23,24]. Descriptive statistics were used to summarize feasibility and engagement data. Two-sample, two-tailed *t* tests were used to assess potential baseline differences in average age, baseline minutes of sleep, minutes of PA and MVPA, hours of video game use, and problematic mealtime behavior scores between the intervention and wait-list control groups. Proportion tests were used to assess potential baseline differences by group in percent male, percent White, percent qualifying for free or reduced-price lunch, and percent taking psychiatric medication associated with weight gain. All analyses were performed in an intention-to-treat manner. Changes in the exercise stage of change by group were assessed using the nonparametric Wilcoxon signed-rank test. Paired *t* tests were used to assess significant differences in pre-post BMI, BP, total PA (light PA+MVPA), MVPA, sleep duration, problematic mealtime behavior score, and hours of nonexergaming video game use by group. STATA 13 was used for all analyses.

## Results

### Overview

Participant demographic information is presented in Table 1. The average participant age was 15.1 years (range 12-17); 74% (17/23) of the participants were male, 40% (9/23) were people of color, and 35% (8/23) qualified for free or reduced-price lunch. ADHD was the most common diagnosis (13/23, 57%), followed by ASD (12/23, 52%), anxiety disorders (6/23, 26%), and depression (6/23, 26%). Nearly one-third (7/23, 30%) of the total sample reported taking psychiatric medication that was associated with weight gain. There were no significant differences in demographic characteristics between the control and intervention groups; however, given the small sample size, it is important to note that 5 out of 12 control participants were reported to be taking medication, whereas only approximately 2 out of 11 intervention participants were reported doing so. In addition, the type of medication is unknown, so the potential directionality of effects on PA, diet, and sleep is unclear (Table 1). However, the average daily MVPA, total PA, exercise stage of change, sleep duration, problematic mealtime behaviors score, and hours of video game use at baseline did not differ significantly between the control and intervention groups.

**Table 1:** Participant characteristics at baseline.

Characteristics		All participants (n=23)	Control group (n=12)	Intervention group (n=11)	Test statistic							*P* value
					*t* test (*df*)^a^		z test		Chi-square (*df*)			
Age (years), mean (SD)		15.1	14.9	15.3	−0.57 (20)		N/A^b^		N/A		.57	
Sex (male), n (%)		17 (74)	10 (83)	7 (64)	N/A		−1.58		N/A		.12	
**Race or ethnicity, n (%)**					N/A		N/A		1.2 (5)		.88	
	Asian	2 (9)	1 (8)	1 (9)								
	Black	2 (9)	1 (8)	1 (9)								
	Hispanic	3 (13)	2 (17)	1 (9)								
	White	14 (60)	7 (58)	7 (64)								
	Declined	2 (9)	1 (8)	1 (9)								
Qualifies for free or reduced-price lunch, n		8 (35)	3 (25)	5 (45)	N/A		N/A		1.5 (1)		.23	
**Diagnoses, n (%)^c^**						N/A		N/A		N/A		N/A
	Autism spectrum disorder	12 (52)	7 (58)	5 (45)								
	Attention-deficit/hyperactivity disorder	13 (56)	8 (67)	5 (45)								
	Anxiety	6 (26)	5 (42)	1 (9)								
	Depression	6 (26)	3 (25)	3 (27)								
	Other	4 (17)	4 (33)	0 (0)								
Taking medication that causes weight gain, n (%)		7 (30)	5 (42)	2 (18)	N/A		N/A		5.5 (2)		.06	

^a^Two-tailed *t* test (age), test of proportion (sex, race or ethnicity, free or reduced lunch, and medication use).

^b^N/A: not applicable.

^c^More than 50% of participants had multiple diagnoses.

### Primary Outcomes

**Figure 2:**
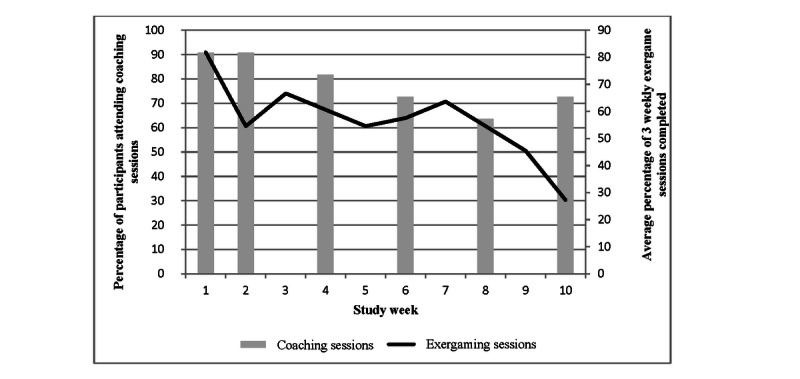
Planned session adherence trends.

**Figure 3:**
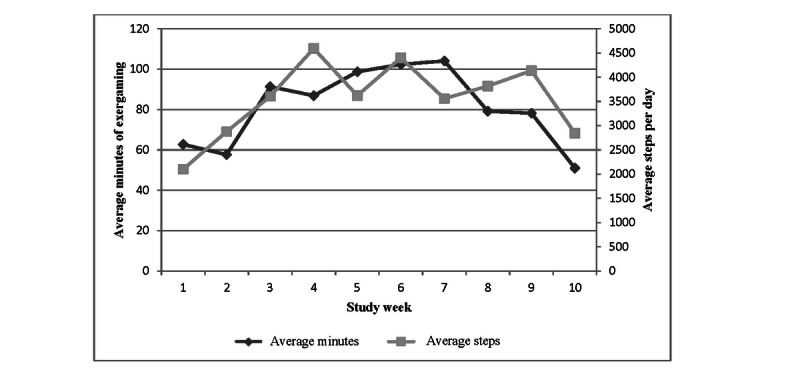
Exercise engagement trends.

Equipment was successfully installed and used by all intervention participants (n=11), who completed an average of 5 out of 6 possible coaching sessions (range 0-6). None of the participants were familiar with the 3 exergames used during the study before the start of the intervention. Including 1 participant who never exergamed or attended coaching sessions after the initial home visit and training session, the intervention group executed an average of 1.7 out of 3 planned exergame sessions per week, averaging 81.2 minutes per week (SD 18.9) of self-reported exergaming and averaging 3559 steps per day over the course of the intervention. Figure 2 shows trends in coaching session and exergame session adherence over time, whereas Figure 3 shows the changes in exercise engagement over the course of the intervention.

Considering the 11 participants, adherence to the coaching sessions exceeded 60% each week—from a high of 10 (91%) attending in weeks 1 and 2 to a low of 7 (64%) attending in week 8 (Figure 2). Exergaming adherence to the prescribed 3 times per week was highest in week 1, with 82% (27/33) of sessions completed; fluctuated between 55% (18/33) and 67% (22/33) in weeks 2 to 8; and then declined to only 27% (9/33) in week 10 (Figure 2). However, the total weekly duration of exergaming increased from about 60 minutes in week 1 to a peak at 104 minutes in week 7, before slowly declining to 51 minutes in week 10 (Figure 3). Finally, PA engagement measured by Fitbit increased from approximately 2100 steps per day in week 1 to 3500-4500 steps per day in weeks 3-9, before dropping to 2850 in week 10 (Figure 3). Of the 9 intervention participants who completed exit surveys, 6 reported intention to continue exergaming, 7 reported enjoying coaching sessions, and 8 reported feeling that the coaching sessions were helpful and that the coach gave them tips they could use. Several caregivers noted during live telehealth sessions that the different technologies involved in the intervention (Wi-Fi, Fitbit, Xbox games, Kinect sensor, and Xbox Skype) presented a significant burden and expressed a desire for a more integrated technology delivery system such as a smartphone app.

The average daily MVPA measured by accelerometry declined between baseline and 1 week postintervention for both the control group (−9.3 min, SD 21.5; *P*=.18) and the intervention group (−1.4 min, SD 8.3; *P*=.62); neither change was statistically significant. However, although average daily total PA (MVPA+light PA) significantly decreased for the control group in postintervention period follow-up (−58.8 min, SD 85.7; *P*=.04), it did not significantly change for the intervention group (−5.3 min, SD 55.1; *P*=.77).

### Secondary Outcomes

Secondary outcomes included pre-post changes in self-reported exercise stage of change, sleep duration, video game use, mealtime behaviors, BMI, and BP. The exercise stage of change improved significantly for the intervention group (*P*=.02) but not for the control group (*P*=.99). None of the treatment participants were in the preparation or action stages before intervention; postintervention, there were 1 participant and 5 participants in the preparation and action stages, respectively. In contrast, although no control group participants were in the preparation stage, 3 were in the action stage before the intervention; postintervention, there were still none in the preparation stage and only 2 in the action stage.

The average daily minutes of sleep decreased by 12 minutes for the control group (*P*=.91) but increased by 12.9 minutes for the intervention group (*P*=.55). The intervention group reported a 1.7-hour decrease in weekly nonexergaming video game use (*P*=.28); the control group reported no change. There were no significant changes in BMI, BP, or problematic meal behaviors in either group.

## Discussion

### Principal Findings

Youth with NPDs are at high risk of unhealthy lifestyle behaviors, including low PA levels [11], poor diet [32], high screen time [10], and poor sleep hygiene [9]. The consequences of these behaviors include elevated chronic disease risks, including obesity; cognitive impairment; and exacerbation of psychiatric symptoms. Barriers to engagement in lifestyle interventions are high among this population [21], and interventions demonstrating long-term engagement in improved health behaviors are scarce. Furthermore, as the COVID-19 pandemic has shown, a remotely delivered intervention using telehealth components can not only reduce barriers to initial participation but also allow flexibility for continued engagement during changing conditions [33], which are particularly important for this population.

The aim of this pilot study is to adapt and expand an existing, evidence-based exergaming and telehealth coaching intervention [16] to improve PA, diet, video game play time, and sleep habits among youth with a variety mental health and neurodevelopmental disorders and to assess the program’s initial feasibility and acceptability, including participants’ engagement in PA. An expert working group made preliminary adaptations, which were then beta tested with the target population; feedback was used to finalize the intervention, and telehealth coaches were trained to use positive reinforcement and behavioral redirection techniques. Youth with relevant diagnoses (n=23) were recruited from both a therapeutic school and clinic setting and randomized to either the 10-week intervention or wait-list control.

Although not as high as the original GameSquad intervention that targeted a younger population (10-12 years vs 12-17 years), compliance with planned exergaming was good (participants completed an average of 57% of planned exergame sessions), and attendance at coaching sessions was excellent (participants attended an average of 5 out of 6 coaching sessions), particularly given the unique barriers faced by participants with NPDs and the technological challenges noted by some caregivers.

Despite the short duration and small sample size that limited our power to detect intervention effects, the results indicated potential improvements to PA during the intervention over baseline and smaller declines in MVPA relative to control participants after the intervention had ended. Engagement in both exergame-based and nonscreen-based PA was good; however, it declined in weeks 9 and 10 of the pilot. When combined with a decline in MVPA in both the control and intervention groups after the intervention was completed, this may reflect a seasonality effect. Such an effect could have been because of the school year ending, discontinuation of physical education classes and school sports, or other external factors. However, the decline in duration of exergame sessions after week 7, combined with qualitative feedback from beta-testing participants, may suggest that the adolescent population in this study became bored with the limited menu of exergames available more quickly than the preadolescent population in the original GameSquad study. Alternatively, it may reflect frustration with or dislike of the increasing intensity demands of the exergaming menu as the intervention progressed. Further exploration of these factors is required to optimize long-term engagement.

Long-term implementation of AGS might reorient coaching to leverage the early weeks of exergame engagement into a greater emphasis on nonscreen-based PA modalities later in the intervention. Additional game choices and technologies should also be evaluated and included to maintain participants’ interest and better meet the needs of this diverse population. The coaching sessions appeared to fill a psychosocial need for participants, independent of exergaming. Compliance with and acceptability of coaching sessions were higher than those of exergaming, with several participants repeatedly rescheduling sessions to enable them to meet with their coaches around significant clinical events such as inpatient hospital stays.

### Secondary Outcome Results

Although the study was underpowered to evaluate secondary outcomes, participants reported increased sleep duration and decreased video game use time, despite the introduction of exergaming sessions. There were no improvements in problematic meal behaviors as a result of participation. Intervention participation also appeared to positively affect the exercise stage of change; although no treatment participants were in the active stage of exercise before the intervention, nearly 50% were in the active stage after the intervention conclusion. It is important to note, however, that although the exercise stage of change improved, the maintenance stage was not evaluated through long-term follow-up.

### Limitations and Additional Research Needs

This pilot study has several limitations. The small sample size limits generalizability and decreased power to detect intervention effects; it also precludes stratified examination of differences in outcomes by subgroup. Although participants were randomized to condition, the sample size was small; thus, as models were not adjusted for potential covariates and confounders, readers need to take caution in interpreting the results. In addition, although the strength of this study was that pre- and post-PA were objectively measured using accelerometers, several other measures were self-reported, including duration of exergaming sessions, sleep duration, and video game use. The 10-week intervention design and lack of long-term follow-up prevented the evaluation of sustained engagement and effects of the intervention; this must be the primary aim of any full-scale efficacy study.

This study has several notable strengths. We were able to include youth with a variety of mental health and neurodevelopmental disorders, recruiting participants from both clinical and therapeutic school settings. We believe this heterogeneity improves external generalizability and eventual translation to a variety of clinical and community venues. Next steps should include the development of a mobile health (mHealth) app to seamlessly deliver a wider variety of exergames, telehealth coaching sessions, and parental and participant reminders and integrate mood, health habit, and PA tracking, while eliminating the technical barriers associated with the Xbox-based approach. This will also help minimize disruptions to upscaling caused by changes in commercially available gaming technologies. Additional research should also be conducted with youth with NPDs to evaluate personal mediators of health behaviors, exercise preferences, and barriers to engagement that may inform mHealth app design and intervention optimization.

### Conclusions

This pilot study is an innovative example of inclusion team science, a term coined by Rimmer and Vanderbom [18] in their 2016 call to action for health promotion research for children with disabilities. Their commentary urged greater collaboration between disability researchers and intervention scientists working with typically developing populations to more rapidly adapt existing interventions to meet the underserved needs of youth with disabilities [18]. In this study, we began with a proven intervention developed for typically developing youth with overweight and obesity and adapted it for youth with a variety of mental health and neurodevelopmental disorders using a process that included beta testing by the target population and adaptation of intervention components and methods based on their feedback. Although our sample size was too small to evaluate efficacy, the initial feasibility and acceptability results indicate that AGS may be a promising avenue for delivering health behavior interventions remotely to youth with NPDs, an increasingly critical need in light of the significant disruptions to in-person learning and clinical care caused by the COVID-19 pandemic. However, it is important to reduce the technological demands of the intervention on caregivers and increase the number and diversity of exergames used in the intervention to sustain engagement by participants. After additional intervention optimization, future efficacy testing must take place in a large, socioeconomically, racially or ethnically, and diagnostically diverse sample of youth.

## Abbreviations

ADHD: attention-deficit/hyperactivity disorder
AGS: Adaptive GameSquad
ASD: autism spectrum disorder
BIS: behavioral inhibition bias
BP: blood pressure
mHealth: mobile health
MIOH: Meals In Our Household Questionnaire
MVPA: moderate- to vigorous-intensity physical activity
NPD: neurodevelopmental and psychiatric diagnosis
PA: physical activity
REDCap: Research Electronic Data Capture
